# Process evaluation of an integrated personalized eHealth programme after total and unicompartmental knee arthroplasty—Results of the multicenter ACTIVE randomized controlled trial

**DOI:** 10.1002/ksa.70319

**Published:** 2026-01-30

**Authors:** A. Carlien Straat, P. Paul F. M. Kuijer, Jelle J. de Jongh, Jantine M. Maarleveld, Judith A. F. Huirne, Gino M. M. J. Kerkhoffs, Johannes R. Anema, Pieter Coenen

**Affiliations:** ^1^ Department of Public and Occupational Health Amsterdam UMC Amsterdam the Netherlands; ^2^ Amsterdam Public Health Research Institute, Societal Participation and Health Amsterdam the Netherlands; ^3^ Amsterdam Movement Sciences Research Institute, Musculoskeletal Health Amsterdam the Netherlands; ^4^ Amsterdam Public Health Research Institute, Societal Participation and Health, Quality of Care Amsterdam the Netherlands; ^5^ Department of Obstetrics and Gynecology Amsterdam UMC, Location VU Medical Center Amsterdam the Netherlands; ^6^ Amsterdam Reproduction and Development Research Institute Amsterdam the Netherlands; ^7^ Department of Orthopaedic Surgery and Sports Medicine Amsterdam UMC, University of Amsterdam Amsterdam the Netherlands; ^8^ Amsterdam Movement Sciences Research Institute, Sports, Ageing and Vitality Amsterdam the Netherlands

**Keywords:** knee replacement, m/eHealth, mobile applications, return to sports, return to work

## Abstract

**Purpose:**

Patients receiving knee arthroplasty often experience limited guidance in resuming daily life activities. The ACTIVE (enhAncing soCietal parTicipation with an IndiVidualized integratEd care programme) trial evaluated the effectiveness of an intervention designed to enhance recovery and return to daily activities in working‐age knee arthroplasty patients. We conducted a process evaluation assessing whether the intervention was delivered as intended and exploring patient experiences, attitudes and the factors that explain how and why the intervention unfolded in practice, to guide future improvements in both the intervention and its implementation.

**Methods:**

A process evaluation was carried out alongside the multicenter randomized controlled ACTIVE trial. Data were analysed using the Linnan and Steckler framework, measuring reach, dose delivered, dose received and patients' attitudes throughout the follow‐up. Among patients in the intervention group only, data were collected using questionnaires, a study database and a logbook of the eHealth programme. We additionally performed six interviews with patients to gather in‐depth information regarding positive and negative experiences with the intervention.

**Results:**

A total of 145 patients were randomized to the intervention group, of which 133 started follow‐up. All components of the intervention were delivered as planned, and 100% of eligible patients received access to the eHealth programme. Adherence was high, with 99% of patients logging into the programme at least once. Patients reported that the intervention was supportive and motivating. The activity tracker was scored highest with a mean of 7.8 (standard deviation [SD] 1.4) and 7.9 (1.4) out of 10 at 6 weeks and 6 months postoperatively, respectively. Patients with disabling comorbidities or atypical recovery trajectories felt that recovery recommendations did not adequately address their specific needs.

**Conclusions:**

The intervention was delivered and used as intended and seems ready for successful implementation for working‐age knee arthroplasty patients with a typical course of recovery. To accommodate patients with atypical recovery trajectories, the intervention should incorporate greater flexibility.

**Level of Evidence:**

N/A.

**Abbreviations**
ACTIVE: enhAncing soCietal parTicipation with an IndiVidualized integratEd care programme
GAS: Goal Attainment Scaling
TKA: total knee arthroplasty
UKA: unicompartmental knee arthroplasty

## INTRODUCTION

After knee arthroplasty, patients are often discharged from the hospital within 1 or 2 days, and usual care after surgery is primarily focused on clinical outcomes such as pain relief and knee function. Additionally, given the current emphasis on cost‐effectiveness in clinical care, usual care increasingly minimizes the length of hospital stay and transfers patient care to the patients' own home. As a consequence, patients experience limited guidance and monitoring on recovery of daily functioning with regard to societal participation [15]. Recommendations for return to daily life activities are often missing [27], and return to work and sport rates are lower than might be expected, considering the pain relief and quality of life improvements after surgery [11, 18, 19, 20, 34]. A way to provide patients with the necessary personalized recovery information after surgery would be to provide them with eHealth programmes at home.

Given the above and because the prevalence of patients receiving a knee arthroplasty is increasing rapidly [1, 12, 16], especially in the working‐age population [13], we have conducted the multicentre ACTIVE (enhAncing soCietal parTicipation with an IndiVidualized integratEd care programme) trial in which we evaluated the (cost‐)effectiveness of an integrated eHealth care programme. The programme aimed to guide return to daily life activities after knee arthroplasty in working‐age knee arthroplasty patients [26] and included a personalized eHealth programme called ‘iRecover’ including an activity tracker, which was supplemented by Goal Attainment Scaling (GAS) and a one‐time case‐manager consultation. We found that the integrated personalized eHealth programme reduced time to return to personal daily life activities compared to usual care [5].

Even though integrated eHealth care programmes have proven to be effective in the past [4, 8, 30, 33], these programmes were not developed for knee arthroplasty patients. In a systematic review, Coenen et al. [6] pointed out that, for orthopaedic surgery patients, a patient‐tailored rehabilitation programme using goal setting and/or eHealth showed small effects on work‐ and/or sports participation post‐surgery. However, implementation of such programmes in practice is not self‐evident. Information on the ‘reach’, ‘dose delivered’, ‘dose received’ and ‘patient attitudes’ is essential for further optimization. Insights into process measures are critical to interpret the outcomes of an intervention and for future scale‐up. The aim of this study was to conduct a process evaluation of our ACTIVE intervention by assessing whether it was delivered as intended and exploring patient experiences, attitudes and the factors that explain how and why the intervention unfolded in practice, to guide future improvements in both the intervention and its implementation.

## METHODS

### ACTIVE trial design

The ACTIVE trial was a multicenter, two‐arm parallel randomized controlled trial with an effectiveness and economic evaluation. This study was approved by the Medical Ethical Committee of Amsterdam UMC, Location VUmc, under registration number NL67692.029.18. It has been registered at the Netherlands Trial Register with reference no. NL8525 (reference date version 1: 14‐04‐2020) and can be found at the International Clinical Trials Registry Platform from the World Health Organization (trialsearch.who.int; reference no. NL8525). The full protocol has been published [26]. ChatGPT (OpenAI) was used solely to improve the grammar and style of the manuscript. OpenAI was not used to interpret scientific content, and all text was verified by the authors. We used the CONsensus‐based Process evaluation reporting guideline for public HEalth intervention Studies (CONPHES) checklist [32] for reporting of this particular process evaluation (Supporting Information S1: Appendix S1). A more specific guideline for reporting of studies in orthopaedics, sport science and rehabilitation [21] was furthermore used to secure relevance for the field.

### Intervention

Patients allocated to the intervention group received, on top of usual care, a transmural integrated care programme aimed at supporting patients in return to self‐chosen daily life activities, including work and sports. The intervention was developed using evidence from the literature [6] and with the input of clinical and scientific experts and patients [24].

The intervention consisted of three components, the details of which can be found in our study protocol [26]. The first component was the personalized iRecover eHealth app that provided patients with recovery recommendations. Approximately 2–4 weeks prior to surgery, patients got access to the app, and patients could select their favourite daily life activities. After surgery, the app informed patients with pop‐up messages on when and how to resume these self‐selected daily life activities. An activity tracker was connected to the app to give patients insight into their physical activity levels during the first 6 months after surgery, and whether a daily step goal was met. Some patients already owned an activity tracker (e.g., a smartwatch). These patients were given the option to either link their personal device to the iRecover application or use the activity tracker provided by the study. In the app, patients completed a recovery monitor once a week (in the first 6 months) and once a month (in the last 6 months), in which they answered which daily life activities they had already resumed and when. Recovery recommendations were altered based on the answers in the recovery monitor, as there were three types of recommendations for each activity: fast, average and slow. Preoperatively, patients were categorized into one of the three recovery rates based on their recovery expectations regarding return to work. Each recovery rate had its own corresponding recovery recommendations: patients in the ‘fast’ recovery rate had more progressive recommendations compared to those in recovery rates ‘average’ and ‘slow’. The second intervention component was a one‐time preoperative case‐manager consultation, during which the app was installed and explained. Also, patient and case manager discussed preoperative recovery expectations. The final component was defining a personalized and work‐related GAS goal. This goal was developed with the case manager, and during the rehabilitation, patients would work on their goal with their own physiotherapist. The intervention lasted either 12 months or until full resumption of daily life activities was achieved [26].

The intervention was compared to usual care. All types of usual care were included and consisted mainly of physiotherapy and pain medication. In the current study, we only analysed data from participants who were randomized into the ACTIVE intervention (not considering the usual care control group participants).

### Participants

Patients within working age (18–67 years) with a paid job for at least eight hours a week, who were on the waiting list for a primary total‐ or unicompartmental knee arthroplasty (UKA) and who were willing to return to work after surgery were eligible to participate in our study [26]. Factors that could affect postoperative recovery in terms of societal participation or factors that might influence the intervention served as exclusion criteria. Patients were recruited from eleven Dutch medical centres (i.e., hospitals and clinics) throughout the Netherlands.

### Data collection

In the current study, we primarily used a quantitative approach. The process evaluation was conducted according to the Linnan and Steckler framework [14]. We identified whether the key components of the intervention were executed according to protocol and/or if our intervention components should be improved for future implementation. We assessed the process measures ‘reach’, ‘dose delivered’, ‘dose received’ and ‘patient attitudes’. Table 1 shows an elaboration of the definitions of these concepts and how they were operationalized in this study. For dose received, we required that participants had logged in so that they would receive their recovery recommendations, and that they had completed at least one recovery monitor, enabling us to determine whether they continued to actively use the app after surgery. Within patient attitudes, barriers to the usage were assessed through statements and questions. Data were collected using information from the study procedure database, a log of the iRecover app and an online patient satisfaction questionnaire that was administered 6 weeks and 6 months after surgery.

**Table 1:** Process measures.

	iRecover eHealth programme	GAS	Case manager
Reach *The degree of participation*	Eligible participants who were (1) interested in participation and (2) were included and randomized to the intervention or usual care control group^a^ as a percentage of the target population. The estimation of the target population was based on the total number of patients who had a knee arthroplasty consultation with an orthopaedic surgeon in the participating centres in 2019, of which 50% were expected to be of working‐age and therefore eligible.		
Dose delivered *The proportion of the intervention that was delivered to the patients*	The percentage of patients in the intervention group who received log‐in details for the iRecover app.^a^	The percentage of patients in the intervention group who were invited to make a GAS goal before surgery.^a^	The percentage of patients in the intervention group who got referred to the one‐time case‐manager consult.^a^
Dose received *The ‘exposure’ and usage of the intervention components*	The percentage of patients in the intervention group that: – Logged in to the iRecover app and filled in their self‐reported favourite activities.^a^ ^,^ ^b^ – Completed at least one recovery monitor.^b^ – Connected their activity tracker to the app.^b^	The percentage of patients in the intervention group who made a work‐related GAS goal before surgery.^a^^,^ b	The percentage of patients in the intervention group who had an appointment with their case manager before surgery.^a^^,^ ^b^
Patient attitudes *Patient satisfaction and barriers for the usage of the intervention*	– The mean (SD) satisfaction score that patients rated the iRecover app on a 1–10^c^ ^,^ ^d^ scale. – The mean (SD) satisfaction score that patients rated the activity tracker on a 1–10^c^ ^,^ ^d^ scale. – Experiences with using the iRecover app.^c^ ^,^ ^d^ ^,^ ^e^	The mean (SD) satisfaction score that patients rated their GAS goal on a scale from 1 to 10.^c^	The mean (SD) satisfaction score that patients rated the case‐manager consultation on a scale from 1 to 10.^c^

Abbreviations: GAS, Goal Attainment Scaling; SD, standard deviation.

^a^ Study procedure database.

^b^ Log of the iRecover app.

^c^ Six‐week questionnaire.

^d^ Six‐month questionnaire.

^e^ Interviews.

For additional in‐depth information, we performed six interviews during May and June 2023 with patients who had completed follow‐up until at least the satisfaction questionnaire at 6 months after surgery. Patients were selected for the interviews if they had given prior permission to be approached for follow‐up research. To ensure that only those who had actively engaged with the intervention were included, we focused on patients who had stated that they had discussed their personal eHealth programme with one of their healthcare professionals (e.g., orthopaedic surgeon, physiotherapists, occupational physician). Furthermore, we used purposeful sampling to make sure we selected patients with different attitudes towards our intervention programme at 6 weeks postoperative based on a scale from 1 (very poor) to 10 (excellent). We recruited two patients who scored lower than 5.5, two patients who scored 6–8 and two patients who scored higher than 8. Interviews were held online using a Microsoft Teams or Zoom video call, conducted by two researchers from the research team (Jelle J. de Jongh with A. Carlien Straat or Jantine M. Maarleveld), and lasted approximately 45 min. Prior to the interviews, the research team held a meeting to design the interview questions. The interview included questions about each intervention component, about barriers and facilitators for usage of the intervention and about patient experiences with each component. Informed consent was obtained before the start of the interview. Audio recordings were made with an Amsterdam UMC secured smartphone and the Philips Voice Recorder mobile app version 4.6.37. All recordings were saved encrypted on the smartphone and deleted directly after exporting it to the Philips SpeechExec Enterprise Transcribe version 7.0 software on secured Amsterdam UMC servers.

### Data analysis

IBM SPSS Statistics 28 was used for analysing the quantitative data, using descriptive statistics, including frequencies with percentages and means with standard deviations. For the qualitative analysis, MAXQDA 2022 software was used. Interviews were transcribed verbatim and pseudonymized by one researcher. To reduce bias, two researchers read and coded the transcripts individually. The transcripts were reviewed for additions to the questionnaire. In‐depth information and quotes were extracted and incorporated in this article.

## RESULTS

A total of 293 patients were included in the ACTIVE trial, of which 287 were randomized into the intervention group (*n* = 145) or control group (*n* = 142). Before randomization, six patients were lost to follow‐up due to a cancelled or rescheduled surgery (*n* = 5) or non‐response (*n* = 1). Figure 1 shows a flowchart of the inclusion and follow‐up process of this study.

**Figure 1:**
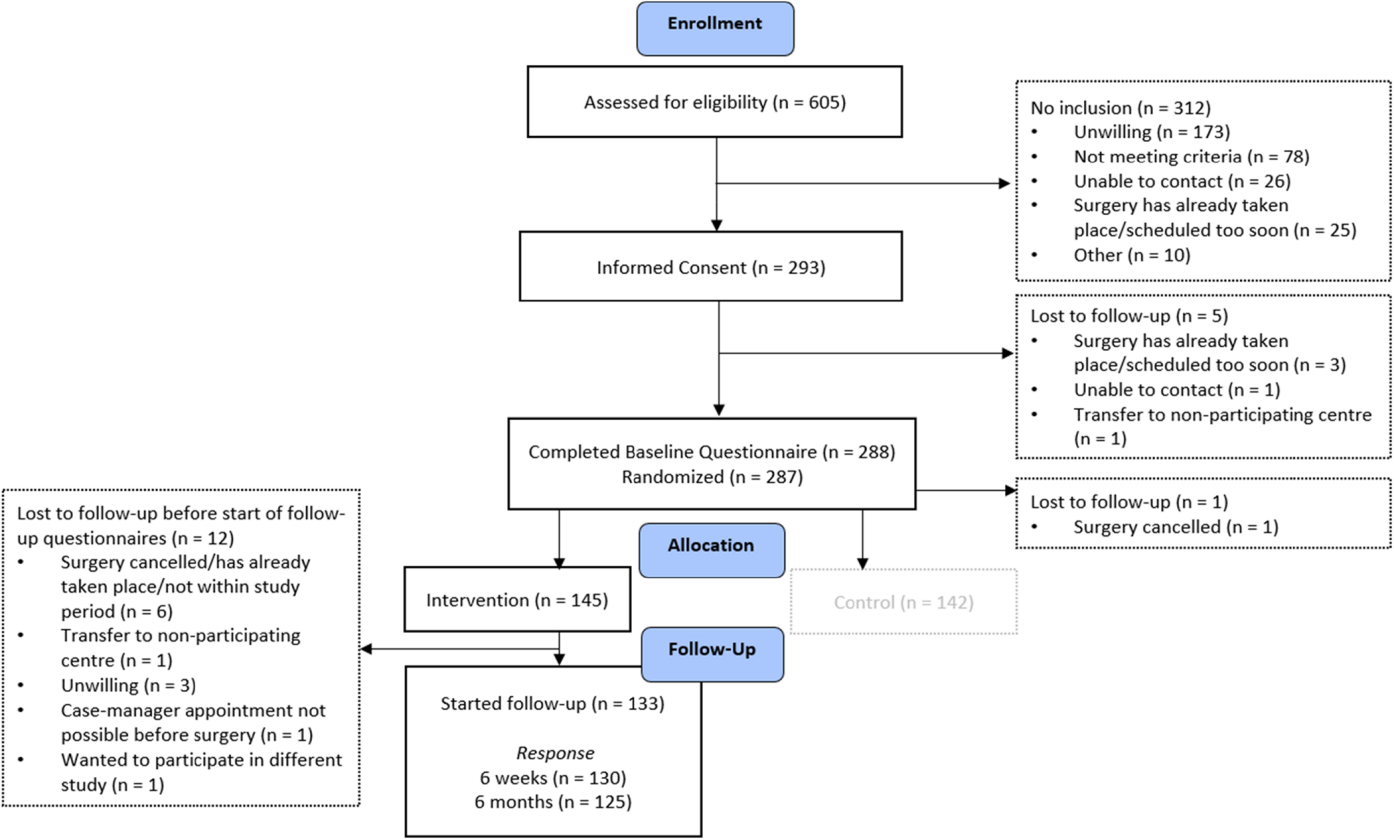
Flowchart of the inclusion, randomization and follow‐up procedure.

### Quantitative process measures

Table 2 shows the results of the quantitative process evaluation conducted according to the Linnan and Steckler framework. Dose delivered was 100% for the three intervention components, that is, all patients who were eligible for our intervention received log‐in details for the iRecover app, were invited to make a GAS goal and got referred to a case‐manager. With regards to the iRecover app, 99% logged in to the app at least once and 97% completed a recovery monitor. Patients demonstrated relatively high monitor compliance (median 87%, interquartile range [IQR]: 59–100). Combining patients who used the study's activity tracker with those who used their own, 94% connected an activity tracker to the app. Concerning GAS, all patients (100%) made a GAS goal; however, 15 (11%) of these goals were not work‐related (which was the intention). The reason for this was that patients did not experience any difficulty with activities at work, hence a work‐related rehabilitation goal seemed not applicable to them. Every patient (100%) had a one‐time appointment with their case‐manager before surgery.

**Table 2:** Results of the process measures by Linnan and Steckler.

	iRecover eHealth programme	GAS	Case manager
Reach *The degree of participation*	In 2019,^a^ the participating hospitals collectively performed a total of approximately 4000 knee arthroplasties [35]. With 50% being of working‐age, an estimated 2000 patients were eligible for our study. This gives a reach of 30% for interested patients (*n* = 605) and 15% for patient inclusion (*n* = 293).		
Dose delivered (*n*, %) *The proportion of the intervention that was delivered to the patients*	133/133 (100%) patients in the intervention group received log‐in details for the iRecover app.	133/133 (100%) of patients in the intervention group were invited to make a GAS goal before surgery.	133/133 (100%) patients in the intervention group got referred to a case manager.
Dose received (n, %) *The ‘exposure’ and usage of the intervention components*	– 132/133 (99%) patients logged in to the iRecover app and filled in their self‐reported favorite activities. – 129/133 (97%) patients completed at least one recovery monitor. – 125/133 (94%) connected their activity tracker to the app.	118/133 (89%) of patients in the intervention group made a work‐related GAS goal before surgery.	133/133 (100%) of patients in the intervention group had an appointment with their case‐manager before surgery.
Patient attitudes *Patient satisfaction and barriers for the usage of the intervention*	– The mean (SD) satisfaction score that patients rated the iRecover app was 7.3 (1.6) at 6 weeks and 7.3 (1.6) at 6 months. – The mean (SD) satisfaction score that patients rated the activity tracker was 7.8 (1.4) at 6 weeks and 7.9 (1.4) at 6 months. – Experiences with using the iRecover app are shown in the paragraphs ‘patient attitudes’ and ‘qualitative results’.	The mean (SD) satisfaction score that patients rated their GAS goal was 7.2 (1.5) at 6 weeks and 6.9 (1.7) at 6 months.	The mean (SD) satisfaction score that patients rated the case‐manager consultation was 7.5 (1.4) at 6 weeks.

Abbreviations: GAS, Goal Attainment Scaling; SD, standard deviation; TKA, total knee arthroplasty; UKA, unicompartmental knee arthroplasty.

^a^ We relied on data from the number of UKA and TKA surgeries performed in 2019, as the Dutch registry reports with a delay, and data from the COVID‐19 years were not considered representative.

### Patient attitudes

On a scale from 1 (very poor) to 10 (excellent) and measured at 6 weeks and 6 months postoperative, patients' attitudes regarding the activity tracker were scored highest, namely, on average 7.8 (standard deviation [SD] 1.4) and 7.9 (1.4), respectively. The appointment with their case manager was scored 7.5 (1.4), measured at 6 weeks only. The iRecover app was scored 7.3 (1.6) at both measurement moments and GAS scored 7.2 (1.5) and 6.9 (1.7) at 6 weeks and 6 months, respectively.

Figure 2 and Supporting Information S1: Appendix S2 show the results from eleven additional questions from the 6‐week and 6‐month patient satisfaction questionnaire on a 5‐point scale with outcome options ranging from ‘strongly agree’ to ‘strongly disagree’. Regarding the iRecover app, 57% of the patients (strongly) agreed that the use of the application positively influenced the resumption of their daily life activities at 6 months, and 50% thought the same regarding resumption of work activities. The activity tracker, according to 80% of the patients, was thought to motivate them to move more at 6 weeks postoperative. This was 82% at 6 months postoperative. Forty‐seven percent of the patients believed that the personal GAS goal facilitated them to rehabilitate more effectively during the first 6 months, while 20% said it did not make a difference for them.

**Figure 2:**
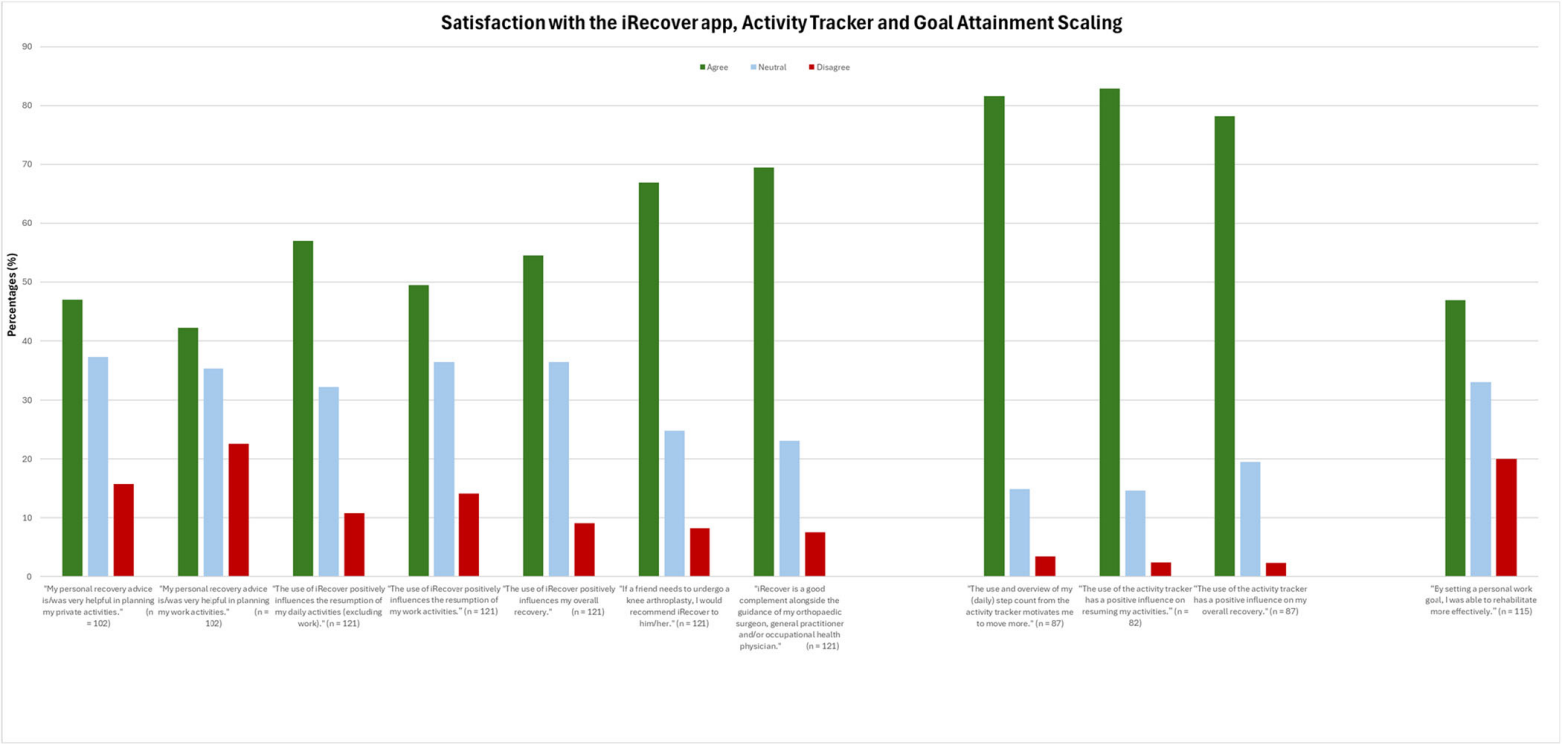
The percentages of patients who were satisfied (agree, neutral, disagree) with the three intervention components—iRecover app, Activity Tracker and Goal Attainment Scaling (GAS) at 6 months postoperative.

### Results from the six patient interviews

Four participants of the interviews were female, and two participants were male. Ages ranged from 56 to 63 years old. From the interviews, several patients found the intervention easy to use. Patients mentioned that the iRecover application and the activity tracker were easy to control, and patients did not experience technical difficulties with the application: ‘I found it very user‐friendly. It ran like clockwork’ (Patient 4, female). The recovery recommendations provided patients with insight and reassured them, and patients liked the availability to check their progress: ‘At the beginning of the recovery, it is very frustrating that you're not able to do almost anything. And after a few weeks or months, if you look back in the app, you can see how much progress you have made’ (Patient 3, female). Also, various patients mentioned that the intervention was perceived as caring: ‘I really liked getting a message almost every day because that made it feel like I was not in this recovery process by myself. Because the day after surgery you leave the hospital and you are kind of expected to rely on yourself’ (Patient 4, female). Patients felt supported and, in most cases, felt as if the intervention was tailored to their recovery and situation: ‘Personally, I think it should be available for everyone in the future’ (Patient 2, female). However, for some patients who experienced a complication, had a comorbidity or a delayed recovery due to other reasons, the intervention was perceived as not sufficiently personalized, as one patient (female) stated in the satisfaction questionnaire: ‘The information is focused on your knee arthroplasty, but does not take other comorbidities into account, such as arthritis in the other knee’. These patients found that the recovery recommendations were not applicable to them, and the algorithm in the app was not flexible enough to change to an alternative recovery speed: ‘There were no options to tell why the recovery was not going as I had hoped’ (questionnaire, female). Also, in the satisfaction questionnaires, some patients stated that they did not discuss their personal recovery programme with their healthcare professionals because they did not think it was necessary or because they did not have an appointment with, for example, their general practitioner or occupational physician.

## DISCUSSION

### Main findings

All components of the intervention were delivered as intended, with 100% of eligible patients receiving access to the iRecover app, setting a GAS goal and having a preoperative consultation with a case manager. Adherence was high: 99% logged into the app at least once, 97% completed a recovery monitor, 94% connected their activity tracker, 89% made a work‐related GAS goal, and all patients had a case‐manager consultation. On a 1–10 scale, the activity tracker received the highest satisfaction ratings compared to the iRecover app, GAS goal and case‐manager consultation. Patients described the intervention as user‐friendly, supportive and motivating. For patients with atypical recovery trajectories, however, the intervention was insufficiently adaptable to their personal situation.

### Interpretation of findings

The process evaluation scores across all components of the Linnan and Steckler model were consistently high. We attribute this to the combination of highly motivated patients, researchers and case managers and the use of previously proven effective intervention elements [4, 8, 10, 30]. Despite the inclusion of patients in eleven different medical centres, the logistics and procedures were the same for each hospital or clinic. This contributed to a uniform implementation process. The case‐manager consultation was a strong start to the intervention, addressing patients' questions or hesitation, and resolving potential technical or information technology (IT) illiteracy barriers for usage of the app. Patients felt that the app was tailored to their unique situation, which we believe played a significant role in enhancing motivation and engagement with the eHealth programme. Unfortunately, some patients with atypical recovery trajectories (e.g., due to comorbidities) described the app as not sufficiently personal, which could explain lower satisfaction ratings for some participants (Supporting Information S1: Appendix S1). It should be acknowledged, however, that although adherence of our intervention to the process measures was high, the proportion of participants who logged into the app more than once or completed more than one monitor was substantially lower. Nevertheless, overall monitor compliance remained relatively high (median >85%). Also, for implementation in routine clinical practice, we recognize that such high levels of patient motivation and interest in eHealth as presented in our study may not be common. Our study population may therefore not be representative of the broader working knee arthroplasty population. Additionally, personal case‐manager consultations may not be feasible in all medical centres. To support broader implementation in usual care, the iRecover application including the activity tracker should function well with limited personalized support from the hospital or clinic, the implementation of which should be studied in future research.

### Comparison with other studies

There are only a limited number of published process evaluations on recovery‐oriented eHealth programmes following surgery, making the comparison of our findings to other similar studies challenging. Within our research group, three process evaluations have been conducted using similar interventions [3, 7, 31]; however, in samples from different patient populations. In those studies, the iRecover eHealth programme was also perceived as supportive during the recovery process. Patients reported that it provided guidance and added value to their postoperative care. Satisfaction scores for both the iRecover app and the activity tracker in our study were comparable with these studies. Our study is the first to incorporate a case‐manager consultation, personalized GAS component and three adaptable recovery speeds into the intervention (allowing for more personalized guidance). Hence, there were no previous data available to compare satisfaction scores regarding these elements.

Among our knee arthroplasty patients and in line with Bouwsma et al. [3], those who were unable to fully adhere to the recovery recommendations generally perceived the eHealth programme as overly optimistic. Within our sample, recovery was generally slower than the provided recommendations [25]. However, Den Bakker et al. [7] reported that when participants did not fully adhere to their personal recommendations, this was primarily due to patients recovering more quickly than expected, making the recommendations feel too conservative. Similar to the findings of Den Bakker et al. [7], some of our participants indicated that the personal recovery recommendations were not sufficiently *adapted to their personal circumstances in case* of disabling comorbidities, atypical recovery courses or specific individual conditions, which was supported by some of the lower quantitative satisfaction ratings. In both studies, this lack of personalization was seen as a significant barrier for the usability, making the intervention less applicable for these patients.

The importance and effectiveness of educating patients have been demonstrated by Timmers et al. [28]. In their systematic review of 21 randomized controlled trials that included 4106 patients, perioperative patient education was shown to be crucial. They found that providing timely information via eHealth improved outcomes such as patient satisfaction and clinical recovery. The authors emphasized the importance of timing, that is, delivering the right information at the right moment. In our study, 60%–83% of patients reported that the iRecover app, the activity tracker or both supported their resumption of daily life activities, suggesting that our intervention successfully provided timely education during recovery.

Finally, we observed in our trial that the development and use of mobile applications during the recovery of knee arthroplasty patients is becoming increasingly popular, at least in the Netherlands. Our intervention focuses on the outcome of return to daily activities, including work and sports, but there are also eHealth programmes targeting clinical outcomes such as pain and knee function. Özden and Sari [17] demonstrated that, compared with conventional rehabilitation, application‐based telerehabilitation yields more effective results in function, pain and range of motion and concluded that this form of care should be considered. Similarly, Dieter et al. [9] showed that their self‐directed, sensor‐based mobile health exercise intervention was superior to usual care for pain reduction. Taken together, these findings suggest that promising eHealth initiatives are emerging in orthopaedics. However, as mHealth/eHealth interventions are complex, requiring significant expertise from both providers and users, the evaluation of such interventions should extend beyond assessing the intended outcomes alone. Following the framework developed by the UK Medical Research Council and the National Institute for Health Research [23], future evaluation studies should address a broader range of questions. These include, for example, identifying additional impacts, theorizing how the intervention works, considering its interaction with the context in which it is implemented and determining how the evidence can inform real‐world decision‐making.

### Strengths and limitations

The design is a strength of our study. We used different sources of information, such as questionnaires, the iRecover app logbook and six in‐depth interviews, so we could perform a complete process evaluation. The systematic data collection was also one of the strengths. During the study period, elaborate study databases and iRecover logbooks were kept and updated with information about each individual patient, ensuring correct and complete data. With regards to the in‐depth interviews, it was a strength that we selected patients with both negative and positive attitudes towards the provided intervention. This ensured representative and both positive and critical feedback from patients with different recovery trajectories. This feedback helped us interpret the quantitative data and provided input for future development, for example, maintaining the flexibility to switch between fast, average and slow recommendations, while also adding the option to adjust advice in case of complications.

This study also has several limitations. First, the target population used to estimate the reach of the intervention is a rough estimate. We relied on data from the number of UKA and total knee arthroplasty (TKA) surgeries performed in 2019 [35], as the Dutch registry reports with a delay, and data from the COVID‐19 years were not considered representative. Additionally, the data did not distinguish between working and non‐working patients, which complicated estimating the actual target population. Therefore, these estimates should be interpreted with caution. Second, the number of patients interviewed was relatively small. This was due to this process evaluation's focus on quantitative data. As a result, we did not perform a full qualitative data analysis and did not aim for data saturation. The in‐depth information we retrieved from the six interviews did probably not provide a complete picture, and the qualitative findings should be interpreted carefully. Third, the amount of time between the interview and active use of the intervention varied for patients. Some patients were included in the trial almost a year before the interview, while others were included only a couple of months earlier. This discrepancy could have resulted in recall bias, as some patients were not able to accurately remember details of the intervention. In addition, to obtain a more comprehensive evaluation of the process, it would be valuable to also investigate the experiences of case managers, orthopaedic surgeons and/or other healthcare professionals involved in the intervention. Finally, all quotes presented in the results section were originally in Dutch and were translated into English by the researchers. Although we aimed to preserve the original meaning and tone, some degree of translation bias cannot be ruled out.

### Implications for future implementation and clinical practice

Based on the results of this study, which demonstrated high adherence and satisfaction, together with the results of our effectiveness analyses showing enhanced return to daily life activities [5], we believe the intervention is ready for implementation. However, further improvements are still possible. It is desirable to adjust the eHealth programme so that the app can better accommodate patients who experience complications or atypical recovery trajectories. The recovery recommendations could be made more flexible, instead of using only the three predefined recovery rates, and include the option to adjust or revert the recovery recommendations to better match the patient's progress. In this way, the app can still be used even when the recovery is not going as expected. Furthermore, integrating the recovery monitor with the patients' own physician or physiotherapist would enhance involvement and allow for early identification of, for example, a delayed recovery. In addition, with integration of clinician feedback, this could improve the flexibility of the recovery recommendations by allowing for adjustments in the recovery recommendations. Ideally, this could help reduce healthcare costs by limiting follow‐up appointments to only those patients with a delayed recovery in the recovery monitor. In addition to this, future versions of our app should pay attention to keeping patients involved with the app, as we observed that limited patients engaged with the app on a daily basis. Nudges, such as challenges or personalized pop‐up messages, could help to enhance engagement.

We are aware that implementation of eHealth in clinical care is challenging [2, 22]. It would be essential to standardize the iRecover app in planning and IT services, while still providing a personalized start to the programme. Ensuring a consistent, personalized and supportive introduction to the programme is critical to achieve similar levels of implementation success in usual care settings. Still, barriers such as limited patient reach, economic concerns or low eHealth literacy can hinder implementation. In addition, scaling up intervention components such as the case‐manager require time and financial resources that medical centres cannot always provide. Toben et al. evaluated a patient‐oriented implementation strategy for m/eHealth and concluded that healthcare professionals should be involved in the process as ‘gatekeepers’, both to provide patients with a sense of security and to help identify those most likely to benefit [29].

## CONCLUSIONS

The integrated personalized eHealth programme was delivered and used as intended and seems ready for successful implementation for working‐age knee arthroplasty patients with a typical recovery trajectory. Patients reported that the intervention positively contributed to their recovery with regard to return to daily life activities, including work and sports. The most frequently mentioned limitation was the limited flexibility of the personal recovery recommendations, particularly in the case of disabling comorbidities or complications.

## AUTHOR CONTRIBUTIONS

A. Carlien Straat, P. Paul F. M. Kuijer, Judith A. F. Huirne, Gino M. M. J. Kerkhoffs, Johannes R. Anema and Pieter Coenen contributed to the conceptualization and design of this study, which was part of a master's thesis by Jelle J. de Jongh. A. Carlien Straat, Jelle J. de Jongh and Jantine M. Maarleveld collected the data. Jelle J. de Jongh together with A. Carlien Straat undertook the analyses and prepared the original draft. These and all other authors provided important intellectual input on the manuscript in various stages of the project.

## CONFLICT OF INTEREST STATEMENT

Judith A. F. Huirne and Johannes R. Anema are advisers of the spin‐off company ikHerstel BV (iRecover) concerning the implementation of the ikHerstel app. Johannes R. Anema holds a chair in Occupational & Insurance Medicine endowed by the Dutch Social Security Institute: the Institute for Employee Benefits Schemes (UWV). The remaining authors declare no conflicts of interest.

## ETHICS STATEMENT

This study was approved by the Medical Ethical Committee of Amsterdam UMC, location VUmc, under registration number NL67692.029.18. Patients were only included in the study upon providing informed consent.

## Supporting information

Appendix 1. CONPHES checklist for reporting of a process evaluation.
Appendix 2. Results from the Patient Satisfaction Questionnaire (with percentages between brackets) at 6 weeks and 6 months.

## Data Availability

After completion of the ACTIVE trial study, we intend to make the data conditionally available through an online repository.
