# Supplementary material for: Process evaluation of an integrated personalized eHealth programme after total and unicompartmental knee arthroplasty—Results of the multicenter ACTIVE randomized controlled trial
Source: Knee Surg Sports Traumatol Arthrosc. 2026 Jan 30;34(10):3691–701. doi: 10.1002/ksa.70319 (PMC13622909; doi:10.1002/ksa.70319)
Supplement: Supplementary file 1 [file KSA-34-3691-s001.docx]

**Appendix 1. CONPHES checklist for reporting of a process evaluation.**

| **#** | **Item** | **Description** |
| --- | --- | --- |
| Title/abstract | | |
| 1 | Title and abstract | Describe in the title and/or abstract and keywords that the study includes a process evaluation |
| Introduction | | |
| 2 | Process evaluation aim(s) and/or objective(s) | Describe aim(s) and/or objective(s) of the process evaluation study |
| Methods | | |
| Intervention | | |
| 3 | Description of the intervention | Describe (1) The intervention development process, with reference to, for example, previous research, frameworks and co-creation that were used in the process of developing the intervention, (2) The intervention, such as the aim of the intervention, components/activities, target population, materials for participants, mode of delivery (eg, face-to-face, online, individual, group-based), duration and frequency and (3) If the intervention was pilot tested. *If applicable, refer to other documents that describe the intervention* |
| 4 | Implementation strategies | Describe implementation strategies (methods or techniques) used to enhance the adoption, implementation and sustainability of the intervention such as materials to support intervention delivery, training of intervention deliverers, organisational support or intervention delivery resources *If applicable, refer to other documents that describe the implementation strategies* |
| 5 | Mechanisms of the intervention | Describe the mechanisms by which the intervention and implementation strategies are expected to achieve the desired effects. *If applicable, present a theory of change or logic model* |
| 6 | Delivery and support team | If applicable, describe who delivered the intervention and implementation strategies, and their expertise such as skills, qualifications and prior experience in delivering the intervention or implementation strategies. *If applicable, describe any potential role members of the research team had* |
| 7 | Context | Describe the context in which the intervention was implemented, such as the type(s) of location(s) or setting(s) where the intervention was delivered, the physical and geographical environment(s), and the socio-cultural and socio-economic context(s) |
| Process evaluation design | | |
| 8 | Process evaluation framework | Describe the theory, model or framework guiding the process evaluation |
| 9 | Process evaluation outcome(s) | Describe any outcome(s) that were assessed in the process evaluation and how they were defined and operationalised. *If assessed, describe process evaluation outcomes such as reach, adoption, recruitment, fidelity, adherence, dosage (delivered and received), quality of delivery, programme differentiation, adaptation, satisfaction, responsiveness, maintenance, costs, facilitators and barriers for implementation* |
| Data collection | | |
| 10 | Recruitment for the process evaluation | Describe how, by whom, and when process evaluation participants (eg, target population, intervention deliverer, location/setting) were recruited, and provide a rationale for the recruitment strategy. *If applicable, describe any eligibility criteria and/or incentives used for participant enrolment.* *If applicable, describe any eligibility criteria for the location(s) or setting(s*) |
| 11 | Process evaluation data collection procedures | Describe (1) What data sources were used (eg, questionnaire, interview, focus group, observation, routine collected data, field notes), (2) Which of the process evaluation outcomes they relate to, (3) the timing of process data collection (eg, at the study start, during and at the end of the process evaluation study) and (4) Data collection setting(s). *If applicable, describe who collected process data, their role in the study and their relationship with study participants (eg, target population, intervention deliverer).* *If applicable, describe whether pre-existing methods and instruments were used or whether they were developed specifically for this study* |
| 12 | Control group data collection | Describe if any process evaluation data were collected among participants in the control or comparison group(s) |
| Analysis | | |
| 13 | Process evaluation data analysis | Describe (1) Data analysis (ie, qualitative, quantitative and/or mixed methods), (2) Who analysed the data and (3) If the results of the effectiveness study were known at the time of process data analysis. *If applicable, name and describe the software used to facilitate the analysis.* *If applicable, describe how data integration techniques were conducted to integrate quantitative and qualitative data.* *If applicable, describe if the relationship between process evaluation findings and effectiveness outcomes was assessed (eg, dose-response analysis*) |
| 14 | Quality assurance of data analysis | Describe techniques that were used to ensure the quality of qualitative and/or quantitative data analysis. *If applicable, for qualitative analyses, describe processes such as member checking, audit trail and triangulation. For quantitative analyses, describe processes such as data cleaning, blinding and how missing data were handled* |
| Results | | |
| 15 | Characteristics of process evaluation participants | Describe characteristics of participants in the process evaluation, and, where relevant, the intervention deliverer(s) and setting(s), and describe to what degree the process evaluation participants and setting(s) were representative of the target population of the effectiveness trial. *If assessed, describe characteristics of the control or comparison group participants* |
| 16 | Results of process evaluation | Describe the results of the process evaluation as defined in the methods section (as listed in items 9 and 13) |
| Discussion | | |
| 17 | Interpretation of findings | Describe how the findings of the process evaluation can be interpreted. *If applicable, describe how effectiveness outcomes should be interpreted in light of the process evaluation findings* |
| 18 | Unexpected factors | Describe previously unforeseen factors that may have influenced the implementation of the intervention and/or the process evaluation results |
| 19 | Implications | Discuss implications of the process evaluation outcomes for future intervention delivery, and implications for policy, practice and/or research |

| **Appendix 2. Results from the Patient Satisfaction Questionnaire (with percentages between brackets) at 6 weeks and 6 months** | | | | | |
| --- | --- | --- | --- | --- | --- |
|  | **Strongly agree** | **Agree** | **Neutral** | **Disagree** | **Strongly disagree** |
| **iRecover application** | | | | | |
| "My personal recovery advice is/was very helpful in planning my private activities."   - 6 weeks (n = 101) - 6 months (n = 102) | 5.0%  8.8% | 43.6% 38.2% | 34.7% 37.3% | 10.9%  11.8% | 5.9%  3.9% |
| "My personal recovery advice is/was very helpful in planning my work activities."   - 6 weeks (n = 101) - 6 months (n = 102) | 5.0%  10.8% | 22.8%  31.4% | 45.5%  35.3% | 17.8%  16.7% | 8.9%  5.9% |
| "The use of iRecover positively influences the resumption of my daily activities (excluding work)."   - 6 weeks (n = 129) - 6 months (n = 121) | 13.2%  14.9% | 47.3%  42.1% | 24.0%  32.2% | 14.7%  9.1% | 0.8%  1.7% |
| "The use of iRecover positively influences the resumption of my work activities.”   - 6 weeks (n = 129) - 6 months (n = 121) | 8.5%  10.7% | 29.5%  38.8% | 43.4%  36.4% | 17.1%  12.4% | 1.6%  1.7% |
| "The use of iRecover positively influences my overall recovery."   - 6 weeks (n = 129) - 6 months (n = 121) | 14.7%  12.4% | 42.6%  42.1% | 34.9%  36.4% | 7.0%  8.3% | 0.8%  0.8% |
| "If a friend needs to undergo a knee arthroplasty, I would recommend iRecover to him/her."   - 6 weeks (n = 129) - 6 months (n = 121) | 22.5%  22.3% | 51.2%  44.6% | 17.8%  24.8% | 7.0%  7.4% | 1.6%  0.8% |
| "iRecover is a good complement alongside the guidance of my orthopaedic surgeon, general practitioner and/or occupational health physician."   - 6 weeks (n = 129) - 6 months (n = 121) | 21.7%  24.0% | 45.7%  45.5% | 20.2%  23.1% | 9.3%  5.8% | 3.1%  1.7% |
| **Activity Tracker** | | | | | |
| "The use and overview of my (daily) step count from the activity tracker motivates me to move more."   - 6 weeks (n = 96) - 6 months (n = 87) | 30.2%  31.0% | 50.0%  50.6% | 14.6%  14.9% | 4.2%  3.4% | 1.0%  0.0% |
| “The use of the activity tracker has a positive influence on resuming my activities.”*   - 6 months (n = 82) | 26.8% | 56.1% | 14.6% | 2.4% | 0.0% |
| “The use of the activity tracker has a positive influence on my overall recovery."*   - 6 months (n = 87) | 27.6% | 50.6% | 19.5% | 2.3% | 0.0% |
| **Goal Attainment Scaling** | | | | | |
| “By setting a personal work goal, I was able to rehabilitate more effectively.”*   - 6 months (n = 115) | 10.4% | 36.5% | 33.0% | 18.3% | 1.7% |
| *This question was not asked at 6 weeks postoperative | | | | | |
| Note: not all patients completed each question, therefore sample sizes are presented and valid percentages are shown. | | | | | |
